# Fluctuations of antibody serum titers for *Toxoplasma gondii* and *Neospora caninum* in naturally infected crossbred cows during gestation

**DOI:** 10.29374/2527-2179.bjvm003023

**Published:** 2023-12-26

**Authors:** Uillians Volkart de Oliveira, Vanessa Carvalho Sampaio de Magalhães, Sônia Carmen Lopo Costa, Ivan Bezerra Allaman, Alexandre Dias Munhoz

**Affiliations:** 1 Veterinarian, Faculdade de Ciências Sociais Aplicadas (FACISA), Itamaraju, BA, Brazil.; 2 Veterinarian, Departamento de Agricultura e Meio Ambiente, Universidade Estadual de Santa Cruz (UESC), Ilhéus, BA, Brasil.; 3 Zootechnics, Departamento de Ciências Exatas e Tecnológicas, UESC, Ilhéus, BA, Brasil.

**Keywords:** crossbred cattle, congenital transmission, toxoplasmosis, neosporosis, gado mestiço, transmissão congênita, toxoplasmose, neosporose

## Abstract

This study aimed to assess the fluctuations of antibody serum titers for *Toxoplasma gondii* and *Neospora caninum* in naturally infected crossbred cows during gestation and to investigate transplacental transmission of *T. gondii*; 51 cows were monitored during pregnancy by monthly serologic testing by indirect fluorescent antibody test. 33 cows were seronegative for both *N. caninum* and *T. gondii*, 10 were seropositive only for *N. caninum*, 5 were seropositive only for *T. gondii,* and 3 were seropositive for both *N. caninum* and *T. gondii*. In both protozoan infections, great variation in antibody levels in pregnant cows was observed, and there was significant increase (p<0.05) in the comparison between the averages of serological titration per trimester. There was a significant correlation (p<0.05) between month and probability of seropositivity for *T. gondii*. We conclude that pregnancy influences antibody titers of crossbred cows naturally infected with *N. caninum* and/or *T. gondii*, and that serologic testing for *T. gondii* in pregnant cows from the sixth month of gestation onward may decrease the number of false negative results.

## Resumo

O objetivo deste estudo foi avaliar a flutuação dos títulos séricos de anticorpos para *Neospora caninum* e *Toxoplasma gondii* em vacas mestiças naturalmente infectadas durante a gestação e investigar a transmissão transplacentária desses protozoários. 51 vacas foram monitoradas durante a gestação, através de sorologia mensal pela Reação de Imunoflorescência Indireta. 33 vacas foram soronegativas para *N. caninum* e *T. gondii*, 10 foram soropositivas somente para *N. caninum*, 5 somente para *T. gondii* e 3 para *N. caninum* e *T. gondii*. Em ambas as infecções, observou-se grande variação nos níveis de anticorpos em vacas gestantes, e houve um aumento significativo (p<0.05) na comparação entre as medias da titulação sorológica por trimestre. Houve correlação significativa (p<0,05) entre os meses e a probabilidade de soropositividade para *T. gondii*. Conclui-se que a gestação influencia os títulos de anticorpos de vacas mestiças naturalmente infectadas por *N. caninum* e/ou *T. gondii* e que testes sorológicos para *T. gondii* em vacas gestantes a partir do sexto mês podem diminuir o número de resultados falsos negativos.

## Introduction

*Neospora caninum* and *Toxoplasma gondii* are obligate intracellular protozoan parasites belonging to the Infra-phylum Apicomplexa. *Neospora caninum* is recognized as an important cause of abortion in cattle worldwide, especially in dairy cattle (Wouda et al., 1998). In contrast, cows are generally resistant to infection by *T. gondii* (Esteban-Redondo & Innes, 1997), even though there are reports of congenital toxoplasmosis and pregnancy loss (Costa et al., 2011; Garcia et al., 2012). However, ingestion of *T. gondii* tissue cysts from infected meat is a important route of infection for humans, with consumption of raw or undercooked meat from infected animals considered a significant public health risk (Tenter, 2009).

*Neospora caninum* and *T. gondii* have canids (Gondim et al., 2004; McAllister et al., 1998) and felids as definitive hosts (Dubey et al., 1970; Frenkel et al., 1970), respectively. The vertical transmission is most important in the epidemiology of *N. caninum* infection in cattle (Bergeron et al., 2000), whereas *T. gondii* has a low rate of transplacental transmission in cattle (Costa et al., 2011; Garcia et al., 2012).

The dynamics of the antibody serum titers for *N. caninum* during of the gestation have been studied mainly in dairy cattle herds composed of pure breed animals (Antonello et al., 2015; González-Warleta et al., 2008; Hasler et al., 2006; Nogareda et al., 2007). To date, few studies have been carried out with bovine herds formed by crossbreed animals (Cardoso et al., 2009).

Experimental studies on variation in serum levels of antibodies against *T. gondii* have been conducted by a little number of researchers (Costa et al., 2011). However, little is known about fluctuation of the antibody serum titers for *T. gondii* in naturally infected animals, during of the gestation, and the percentage of congenital transmission of the parasite in cattle infected before of gestation. The understanding of these aspects is important, since *T. gondii* DNA has already been identified in seronegative cattle (Opsteegh et al., 2011) and calves (Wiengcharoen et al., 2011).

In light of the above mentioned, the present study aimed at assessing fluctuations of the antibody serum titers for *T. gondii* and *N. caninum* in naturally infected crossbred cows during gestation, and to compare transplacental transmission of *T. gondii* with previous results in the same herd for *N. caninum* in cattle infected before of gestation.

## Materials and methods

### Study site

The study was carried out in the county of Ibicaraí, State of Bahia, northeast Brazil, which annual rainfall is 1,800mm with an annual relative humidity of 80% and an average temperature of 24ºC. This county is located in the Ilhéus-Itabuna microregion (altitude of 47m, Latitude South 14º70', and Longitude West 39º03'). The research proposal was approved by the Committee for Animal Research Ethics (protocol no. 0030/2018) at State Santa Cruz University (UESC), Brazil.

### Selection of farms

The present study was conducted in a farm with a history of cattle being exposed to *N. caninum* in the environment (Galvão et al., 2011; Magalhães et al., 2014). The bovine herd from the farm selected for this seroepidemiological survey consisted of crossbreed animals (¼ Zebu, ¾ European to ¼ European, and ¾ Zebu). The breeding system was semi-intensive, with cattle were supplemented daily with concentrate in a trough.

It was a 135-hectare premise with a total of 350 heads. On average, 110 were lactating cows with a production of 1,200 liters of milk per day. These animals were fed through rotational grazing with mineral salts ad libitum in the trough. Cattle also received concentrate based on corn, soybean, and urea. Cows were milked twice daily using a mechanical milking machine in a closed system.

### Sample collection

Fifty-one cows (10 seropositive only for *N. caninum*, 5 seropositive only for *T. gondii,* 3 seropositive for both *N. caninum* and *T. gondii*, and 33 seronegative for both *N. caninum* and *T. gondii*) were monitored throughout all pregnancy by periodic serologic testing repeated monthly (each 30 days) in order to determine antibody serum titers for these two protozoan infections. In the present study, the inclusion and identification of seropositive and seronegative cows was performed using blood samples of these same animals that were collected in three moments (at intervals of 3 to 6 months) before the start of this study (Magalhães et al., 2014). The animal was considered negative when all three samples were negative. All cows were artificially inseminated, and pregnancy was diagnosed by rectal palpation.

Blood samples were collected by coccygeal venipuncture from cows and jugular venipuncture from calves using 10mL Vacutainer® siliconized glass tubes without anticoagulant. Blood samples were stored in an isothermal container with recyclable ice until routine laboratory processing.

After clot retraction, blood samples were centrifuged at 350*g* for 10 minutes to obtain serum samples. These serum samples were packed in 2.0mL plastic cryotubes and stored at -20°C until serologic testing was performed.

### Serologic testing

The indirect fluorescent antibody test (IFAT) was performed for *N. caninum* and *T. gondii* in serum samples according to the protocols published by Yamane et al. (1997) and Camargo (1964), respectively. IFAT slides were sensitized using tachyzoites of the NC-BA strain (Gondim et al., 2001) for *N. caninum* and of the RH strain for *T. gondii*. A bovine anti-IgG conjugate (Sigma-Aldrich F4387 inc., USA) was used as the secondary antibody. The cut-off point used for *N. caninum* infection was 1:200 for cows (Dubey et al., 1997) while the cut-off point for *T. gondii* infection used both for cows was 1:64 (Costa et al., 2011; Garcia et al., 1999). The negative and positive controls used in this study were those previously used in the studies conducted by Galvão et al. (2011).

### 2.5 Statistical analysis

A mixed linear model was used in all analyses considering the cow effect as random. When the titration was considered as a continuous quantitative variable, the Gaussian distribution was used. When the titration was considered as a discrete quantitative (negative - 0 and positive - 1) the binomial distribution was used.

In the continuous quantitative approach, when months were considered as a quantitative factor, a regression analysis was used. When months were considered as a qualitative factor (grouping the months into quarters) the Tukey test was performed with a significance level of 5%.

In the discrete quantitative approach, a logistic regression was used in addition to the odds ratio. A 95% confidence interval was determined for the odds ratio.

All analyses were performed using a R software (R Core Team, 2015) with the help of the lme4 (Bates et al., 2015) and lsmeans (Lenth, 2016) packages. Animals that tested negative by IFAT had their titer considered zero.

## Results

There were large fluctuations of the antibody serum titers for both *N. caninum* and *T. gondii* in pregnant cows (Tables 1 and 2). During all stages of pregnancy five cows had detectable titers for *N. caninum* (Table 1) whereas only two cows that had detectable titers for *T. gondii* (Table 2); 15.38% (2/13) cows seropositive for *N. caninum* and 62.5% (5/8) cows seropositive for *T. gondii*, respectively, seroconverted to these protozoan organisms during gestation. All 33 seronegative cows remained negative for both pathogens by IFAT at different stages of gestation.

**Table 1:** Fluctuation of anti-*Neospora caninum* antibody titers during gestation of crossbred cows in Ibicaraí, Bahia, Northeast Brazil.

**Cow**	**1st** 1	**2nd**	**3th**	**4th**	**5th**	**6th**	**7th**	**8th**	**9th**
**338**	Neg^2^	Neg	Neg	Neg	Neg	Neg	Neg	800	200
**460**	400	400	400	1600	1600	800	400	800	Neg
**329**	400	200	800	1600	1600	1600	1600	1600	800
**82**	400	400	400	400	800	3200	800	1600	800
**136**	200	400	400	200	800	800	800	400	Neg
**299**	800	200	400	400	800	800	400	800	400
**475**	Neg	Neg	Neg	Neg	800	800	1600	800	800
**066**	200	200	200	200	200	200	1600	400	800
**100**	----3	200	400	800	400	800	800	400	1600
**97**	200	200	200	400	400	400	400	1600	800
**174**	800	400	400	1600^4^	------	----	----	----	----
**212**	800	1600	1600	1600	1600^4^	----	----	----	----
**508**	800	800	1600	1600	800	800	1600^4^	400	----

1 Month of pregnancy;

2 IFAT <200;

3 Uncollected blood;

4 Abortion.

**Table 2:** Fluctuation of anti-*Toxoplasma gondii* antibodies during gestation of crossbred cows in Ibicaraí, Bahia, northeast Brazil.

**Cow**	**1st** 1	**2nd**	**3th**	**4th**	**5th**	**6th**	**7th**	**8th**	**9th**
**246**	256	256	Neg^2^	Neg	Neg	128	256	64	256
**299**	Neg	Neg	Neg	Neg	Neg	Neg	128	Neg	128
**460**	Neg	Neg	Neg	Neg	Neg	256	256	64	256
**338**	Neg	Neg	Neg	Neg	Neg	Neg	64	64	64
**298**	64	64	64	64	128	64	256	256	256
**104**	Neg	Neg	128	512	256	Neg	512	256	128
**200**	256	256	128	128	512	512	256	256	512
**348**	Neg	Neg	Neg	Neg	Neg	Neg	256	256	256

1 Month of pregnancy;

2 IFAT <64.

As pregnancy progressed, a significant increase (p<0.05) was observed in the comparison between the averages of serological titration per trimester for both *N. caninum* (between the 1st trimester and the 2nd and 3rd trimesters) and *T. gondii* (between the 1st and 3rd trimesters) as shown in Table 3.

**Table 3:** Mean quarterly of anti-*Neospora caninum* and *Toxoplasma gondii* antibody titers and the 95% confidence interval during gestation of naturally infected crossbred cows in Ibicaraí, Bahia, northeast Brazil.

***N. caninum***			***T. gondii***			
Quartely	Mean	Lower limit	Upper limit	Mean	Lower limit	Upper limit
T1	448.22a	126.42	770.02	61.33a*	-22.10	144.76
T2	812.70b	477.33	1148.07	114.67ab	31.24	198.10
T3	859.72b	511.91	1207.54	210.24b	127.24	294.10

* Means followed by same letter do not differ in columns (p <0.05) by the Tukey test.

A significant correlation (p<0.05) between months and the probability of an animal being seropositive was observed only for *T. gondii* infection. Therefore, it can be inferred that the chances of an animal being seropositive increases by 2.12 times each month of gestation as opposed to remaining seronegative.

## Discussion

In this study, there was an increase in the antibody titers for *N. caninum* in cows during gestation. This finding corroborates the ones from other studies on bovine neosporosis carried out by other researchers on purebred dairy cows (Antonello et al., 2015; González-Warleta et al., 2008; Hasler et al., 2006; Nogareda et al., 2007) and in crossbred cows (Cardoso et al., 2009). Similar results were obtained in the antibody titers for *T. gondii* which suggest recrudescence of infection due a possible hormonal influence during pregnancy. All animals in this study shared the same pasture and no previously negative animals (both *T. gondii* and *N. caninum*) seroconverted throughout the study, validating our inclusion criteria and allowing us to serve as environmental control. Costa et al. (2011) found no significant difference (p>0.05) in the gestation trimesters in cattle experimentally infected with *T. gondii* at the beginning of pregnancy, demonstrating a difference in the dynamics of antibody fluctuation between experimentally infected animals and naturally infected animals.

For *N. caninum*, most cows remained with detectable titers throughout pregnancy. This result corroborate those published by Cardoso et al. (2009) and Antonello et al. (2015), and differ from the antibody titers observed for *T. gondii* infection in which the detection occurred mainly in the 6th month of gestation. These findings stand in contrast with the findings published by Macedo et al. (2012a) and Garcia et al. (2012). These authors did not find significant differences (p>0.05) in the number of positive cows naturally infected with *T. gondii* that were slaughtered at different stages of pregnancy. However, the cows from these studies were not tested during throughout the entire gestation period, which precludes a more precise analysis of fluctuations of antibody titers.

In the present study, it was observed that, as pregnancy progressed, cows were more likely to become seropositive for *T gondii*. This finding is in agreement with the findings published by Macedo et al. (2012a) who observed that pregnant cows were 8.4 times more likely to be seropositive for *T. gondii* than non-pregnant cows. These results may be important from an epidemiological standpoint. The selection of pregnant cows in the sixth month of gestation for prevalence studies could decrease the number of false negatives (Macedo et al., 2012b) since the antibody titers usually found for *T. gondii* in cattle are low (Costa et al., 2011; Garcia et al., 2012; Macedo et al., 2012a,b).

A low number of seropositive calves for *T. gondii* were detected in our study. This finding corroborates with those from studies carried out in cows of undetermined breeds in natural conditions (Costa et al., 2011). However, there are reports of positive calves in bioassay, but seronegative. These results demonstrate that using serology alone may underestimate the vertical transmission (Macedo et al., 2012b). The results of Costa et al. (2011) and Garcia et al. (2012) demonstrated that vertical transmission occurred mainly in fetuses with at least seven months, suggesting that although rare, when transmission occurs, it is more frequent in the last trimester of gestation. These results coincide with the timing of the increase in antibody titer observed in our study.

The high rate (100%) of transplacental transmission for *N. caninum* in the same herd this study (Magalhães et al., 2014) demonstrates that transplacental transmission for *N. caninum* was more efficient than for *T. gondii* in animals with the same type of management. These findings reinforce that the main mechanism of infection of cattle by *T. gondii* is through horizontal transmission (oocyst ingestion).

In the present study, there were 3 abortions and 1 stillborn in cows seropositive for *N. caninum*. Although it is possible that abortions occurred due to *N. caninum* infection (Dijkstra et al., 2002; Kul et al., 2009), the lack of fetal serology, PCR, histopathology, and immunohistochemistry precludes a definitive diagnosis (Munhoz et al., 2011). In this study, cows seropositive for *T. gondii* did not abort. This finding corroborates the ones published by Costa et al. (2011) and Cabral et al. (2013) and shows that economic losses due to abortion are not expected in cattle infected with *T. gondii* except when cows are exposed to virulent strains (Wiengcharoen et al., 2011) with onset of abortion (Gottstein et al., 1998).

## Conclusion

Based on our findings the serological evidence suggests that: pregnancy influences antibody titers of crossbred cows naturally infected with both *N. caninum* and with *T. gondii*; vertical transmission of *T. gondii* is low*;* and serology of cows in the sixth month of pregnancy can decrease the number of false negatives for *T. gondii*.
